# Experimental liver fibrosis research: update on animal models, legal issues and translational aspects

**DOI:** 10.1186/1755-1536-6-19

**Published:** 2013-10-01

**Authors:** Christian Liedtke, Tom Luedde, Tilman Sauerbruch, David Scholten, Konrad Streetz, Frank Tacke, René Tolba, Christian Trautwein, Jonel Trebicka, Ralf Weiskirchen

**Affiliations:** 1 Department of Internal Medicine III, RWTH University Hospital Aachen, Aachen, Germany; 2 Department of Internal Medicine I, University Hospital Bonn, Bonn, Germany; 3 Institute of Laboratory Animal Science, RWTH University Hospital Aachen, Aachen, Germany; 4 Institute of Clinical Chemistry and Pathobiochemistry, RWTH University Hospital Aachen, Aachen D-52074, Germany

**Keywords:** Animal models, Animal welfare, Cholestasis, Cirrhosis, EU-Directive 2010/63, Fibrosis, Hepatic stellate cells, Hepatocellular carcinoma, Liver immunology, Translational medicine

## Abstract

*Liver fibrosis* is defined as excessive extracellular matrix deposition and is based on complex interactions between matrix-producing hepatic stellate cells and an abundance of liver-resident and infiltrating cells. Investigation of these processes requires *in vitro* and *in vivo* experimental work in animals. However, the use of animals in translational research will be increasingly challenged, at least in countries of the European Union, because of the adoption of new animal welfare rules in 2013. These rules will create an urgent need for optimized standard operating procedures regarding animal experimentation and improved international communication in the liver fibrosis community. This review gives an update on current animal models, techniques and underlying pathomechanisms with the aim of fostering a critical discussion of the limitations and potential of up-to-date animal experimentation. We discuss potential complications in experimental liver fibrosis and provide examples of how the findings of studies in which these models are used can be translated to human disease and therapy. In this review, we want to motivate the international community to design more standardized animal models which might help to address the legally requested replacement, refinement and reduction of animals in fibrosis research.

## Review

### Current concepts in liver fibrosis research

**Figure 1:**
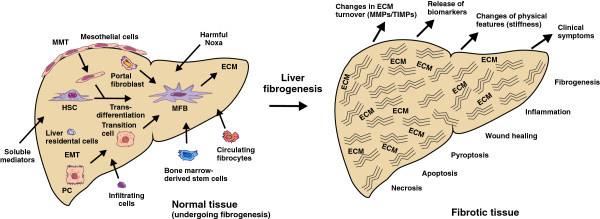
**Pathogenetic concepts in hepatic fibrogenesis.** Hepatic fibrogenesis is a complex reaction that is triggered by many different noxa, including viruses, alcohol and drugs. At the cellular level, liver residential cells (hepatic stellate cells (HSCs) and portal fibroblasts) and infiltrating profibrogenic cells (PCs; circulating fibrocytes and marrow-derived stem cells) cause the formation of excess production and deposition of extracellular matrix (ECM) components. The pool of fibrogenic cells is further increased by epithelial-to-mesenchymal transition (EMT), in which nonparenchymal epithelial cells transition into mesenchymal cells, and further by mesothelial-to-mesenchymal transition (MMT), in which mesothelial cells from the organ surface migrate into the inner part of the liver and acquire a mesenchymal phenotype. In the fibrotic liver tissue, the turnover of the ECM is changed, several biomarkers are released, physical features (stiffness) are altered and clinical symptoms that are characteristic of liver insult develop. MFB, myofibroblast; MMP, matrix metalloproteinase; TIMP, tissue inhibitor of metalloproteinase.

Fibrosis and cirrhosis are both strictly defined pathological entities that were broadly defined by pathologists and hepatologists several decades ago [1,2]. *Cirrhosis* is a diffuse process characterised by fibrosis and the conversion of normal liver architecture into structurally abnormal nodules that affect the whole organ [1]. *Fibrosis* is defined as the presence of excess collagen due to new fibre formation that causes only minor clinical symptoms or disturbance of liver cell function [1]. However, disease-associated abnormalities, including portal hypertension, might be caused by fibrosis alone, depending on its location within the liver [1]. Although hepatic fibrosis in humans can be caused by various stimuli (congenital, metabolic, inflammatory, parasitic, vascular, toxins or drugs), the molecular mechanisms underlying fibrosis are basically the same [3]. Following liver injury of any kind, a defined program of molecular changes occurs that is highly orchestrated at the cellular and molecular levels [4]. This process is characterized mainly by cellular activation of hepatic stellate cells (HSCs) which acquire a myofibroblast (MFB) phenotype and are able to express and deposit large quantities of extracellular matrix (ECM) components within the liver [5,6]. If the insult is temporarily, these changes are transient and liver fibrosis may resolve. If the injury is sustained, however, chronic inflammation and accumulation of the ECM persist, leading to progressive substitution of normal liver parenchyma by scar tissue. In this scenario, the pool of matrix-producing cells is further enlarged by other precursors of MFBs that are recruited from portal fibroblasts and circulating bone marrow–derived, fibroblast-like cells, termed *fibrocytes*. These cells are attracted by soluble mediators within the injured organ, and all contribute to the massive ECM within the affected organ (Figure 1). As a consequence, the composition of the ECM in the injured tissue is altered in regard to quantity and quality from the physiological matrix [4]. In the pathogenesis of chronic liver disease, ECM homeostasis is further disturbed by an unbalanced activity of matrix metalloproteinases (MMPs) and their tissue inhibitors (TIMPs). MMPs represent a large family of zinc- and calcium-dependent enzymes that are responsible for the degradation of ECM proteins. Activated HSCs and MFBs have been identified as prominent cellular sources of MMPs and TIMPs [7]. The combination of various MMPs and TIMPs depends on the disease phases and results at later stages of liver injury in an expression pattern in which MFBs express a combination of MMPs that have the ability to degrade normal liver matrix while inhibiting degradation of the fibrillar collagens that accumulate in liver fibrosis [8].

Moreover, investigators have shown that epithelial cells (that is, hepatocytes, cholangiocytes or other hepatic progenitors) can transition into mesenchymal cells in a process termed *epithelial-to-mesenchymal transition* (EMT) [9]. Although the hypotheses regarding the underlying mechanisms of this process are presently controversial [10-12], the mechanisms might reflect clear differences in cellular behaviour *in vitro* and *in vivo*[13]. Although this exciting discussion of EMT is ongoing, a recent study proposed that mesothelial cells also have the potential to transition into mesenchymal fibrogenic cells via a mechanism called *mesothelial-to-mesenchymal transition* (MMT) [14]. Although this concept is extremely challenging and adds a good explanation of the occurrence of cellular heterogeneity, deeper insights into the precise mechanisms leading to MMT are mandatory to estimate their impact on hepatic fibrogenesis. Diseased organs that undergo fibrogenesis are marked by the simultaneous existence of inflammation, apoptosis, necrosis, pyroptosis and wound-healing. Fibrogenesis results in clinical symptoms, changes in physical features of the liver and release of biomarkers that are directly or indirectly linked to the inflammatory or fibrotic activity within the liver (Figure 1).

Experimental studies that were conducted in isolated primary hepatic cells and experimental animal models led to the identification of general pathogenetic mediators––signalling pathways that are involved in the fibrogenic response. Aberrant activity of transforming growth factor β1 (TGF-β1) or members of the platelet-derived growth factor family are the most prominent drivers of cellular activation and transdifferentiation of HSCs into MFBs [4]. In addition, several chemokines that are released by diverse infiltrating cell populations modulate the inflammatory reaction and contribute to the progression of HSC activation and the fibrotic insult [15], demonstrating the complexity of the disease process. Some of the temporal sequences of molecular events associated with HSC activation can be appropriately reproduced in primary HSC cultures or even in immortalized cell lines [16]. Cell lines are prone to genotypic and phenotypic drift at high passage numbers, however, and are definitely not suitable for mimicking the complex cellular dynamics of HSCs in primary culture. On the basis of this fact, it is obvious that all experimental findings have to be critically evaluated in suitable models that reflect the pathogenetic mechanisms of human hepatic disorders before they can be translated into routine clinical treatments. Therefore, meaningful findings with biological relevance can only be determined in primary cells or, even better, in the *in vivo* context with acceptance of an ethical framework.

**Figure 2:**
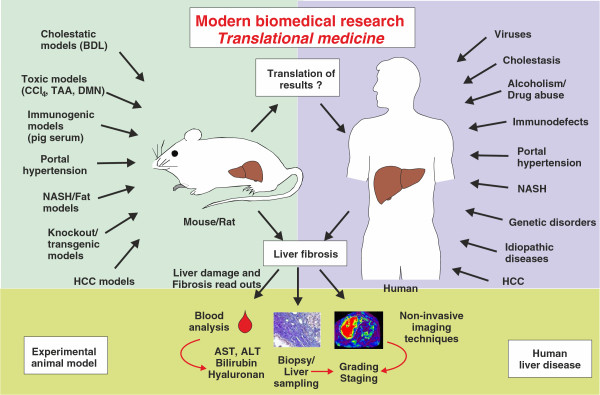
**Translational aspects of fibrosis research.** In hepatology research, diverse cholestatic, toxic, immunogenic and knockout/transgene models, as well as models for portal hypertension, hepatocellular carcinoma (HCC) and fatty liver disease, are presently used. In all of these models, disease progression is associated with hepatic fibrogenesis. These models are suitable to reflect human liver disease of any aetiology. In both the experimental setting (animals) and the clinical setting (humans), the readout systems used to assess hepatic fibrosis are based on blood analysis, histocytochemical analysis and noninvasive imaging techniques. AST, aspartate aminotransferase; ALT, alanine aminotransferase; BDL, bile duct ligation; CCl_4_, Carbon tetrachloride; DMN, Dimethylnitrosamine; NASH, Nonalcoholic steatohepatitis; TAA, Thioacetamide.

In fibrosis research, experimental work in rodents is presently the gold standard to confirm a proposed disease-associated mechanism and specialized protocols that should closely mimic one or the other clinical situation (Figure 2). Moreover, readout systems for liver insults are similar, and sometimes even identical, in humans and animals and include blood tests, biopsy and noninvasive imaging techniques. However, the findings obtained by using these methods may vary between different laboratories and are influenced by the institutional or country-specific stipulations under which respective experiments are performed. Therefore, a Gold Standard Publication Checklist (GSPC) for animal studies was recently proposed that should reduce the number of animals used, lead to more reliable outcomes of animal studies, improve the overall quality of scientific papers based on animal experimentation and follow the idea of evidence-based medicine in science [17]. In addition, it is self-evident that more precise international standards and guidelines that would reduce the overall experimental variation and increase the methodological quality of animal research would further contribute to refinement and reduction of animal experimentation and better translate the findings observed in respective models to the clinic. These intentions were started in 1959, when Russell and Burch proposed an ethical framework for conducting scientific experiments with animals that is based primarily on the replacement, refinement and reduction (3R) principle [18]. This ethical framework has been the subject of intensive debate in which viewpoints shifted significantly during the 20th century [19-21]. As a consequence of all these debates, all member states of the European Union (EU), for example, have to implement EU-Directive 2010/63 regarding the protection of animals used for scientific purposes in 2013.

In this review, we summarize current animal models that are in use and describe the mechanisms that underlie the formation of hepatic fibrogenesis. We discuss basic necessities that will affect fibrosis research in accordance with the new European Animal Welfare Rules that will be implemented at the end of 2013.

## Current animal models in liver fibrosis research

### Cholestatic models of liver injury

Cholestatic liver injury is one of the major causes of liver fibrosis and cirrhosis in patients with acute or chronic liver disease. Damage to the biliary epithelium and bile duct injury can lead to end-stage liver disease, liver failure, organ transplantation or death. The clinical characteristics of this condition are cholestasis, inflammation and liver fibrosis. Multiple causes of bile duct injury have been described. These include autoimmune diseases (that is, primary biliary cirrhosis (PBC) and primary sclerosing cirrhosis (PSC)), obstructive conditions (cholelithiasis and tumour compression of bile ducts) and toxic injury (drugs, chemicals and detergents). To analyse the pathophysiologic processes leading to cholestatic liver injury, animal models mimicking these multiple specific conditions have been generated in the past. These mouse models often focus on specific causes of cholestatic liver disease, such as bile duct obstruction and autoimmune or direct toxic injury.

Surgical bile duct ligation (BDL) is the most common model used to induce obstructive cholestatic injury in mice and rats. Typically, a midsection laparotomy is performed while the animals are under deep anaesthesia, and the common extrahepatic bile duct is ligated twice and dissected. After 21 to 28 days, mice and rats develop jaundice and a strong fibrotic reaction originating from the periportal fields [22]. Different operation techniques have been described for special study settings. Special operating procedures allow reconnection or reanastomosis after bile duct ligation [23]. Other techniques have been described, such as partial BDL [24] or microsurgical methods [25]. This model allows a fast and reproducible way to inflict cholestatic liver injury. Furthermore, this model can be used in transgenic mice easily, allowing the investigation of cholestatic injury in many different study designs.

In recent years, many genetically modified mouse models used to study chronic cholestasis and/or autoimmune liver fibrosis have been described. Genes altered in these mice include the multi-drug-resistant gene 2 (*MDR2*), transforming growth factor β receptor type IIa (*Tgfbr2*), interleukin 2Rα (*Il2ra*), *Ae2*_*a,b*_ and *NOD.c3c4*. MDR2 in mice and MDR3 in humans are class III multi-drug-resistant P-glycoproteins which act as canalicular phospholipid translocators and are involved in biliary phospholipid (phosphatidylcholine) excretion. In humans, mutations in the *ABCB4* gene encoding MDR3 are usually associated with the loss of canalicular MDR3 protein and/or the loss of protein function. These mutations are associated with low biliary phospholipid levels, resulting in a high biliary cholesterol saturation index. Accordingly, several human diseases are linked to mutations of the *ABCB4* gene (progressive familial intrahepatic cholestasis, low phospholipid–associated cholelithiasis syndrome, intrahepatic cholestasis of pregnancy, drug-induced liver injury, transient neonatal cholestasis and adult biliary fibrosis) [26].

Likewise, an *MDR2* (*Abcb4*) gene knockout in mice results in a deficiency in excretion of phosphatidylcholine into bile. Low biliary phospholipid levels trigger nonpurulent inflammatory cholangitis with portal inflammation and ductular proliferation beginning shortly after birth and progressing to end-stage disease in the course of 3 to 6 months. The animals develop a phenotype resembling sclerosing cholangitis with biliary fibrosis and hepatocellular carcinoma [27].

Transgenic mice overexpressing a dominant-negative TGF-β receptor restricted to T cells (*dnTGFβRII* mice) develop an inflammatory biliary ductular disease that strongly resembles human PBC [28]. Next to a spontaneous production of antimitochondrial antibodies (AMAs) directed to the same mitochondrial autoantigens as in human disease (for example, the E2 component of the pyruvate dehydrogenase complex (PDC-E2), the E2 subunit of the branched chain 2-oxo-acid dehydrogenase complex (BCOADC-E2) and the E2 subunit of the 2-oxo-glutarate dehydrogenase complex (OGDC-E2)), these mice show a lymphocytic liver infiltration with periportal inflammation similar to histological changes in human PBC.

Another murine model for human PBC is a knockout mouse strain lacking the interleukin 2 receptor, α chain (IL2Rα) gene. These mice spontaneously develop portal inflammation and biliary ductular injury similar to that of human patients. Portal cell infiltrates show many CD4^+^ and CD8^+^ T cells and increased levels of interferon γ (IFN-γ), tumour necrosis factor α (TNF-α), IL-2 and IL-12p40, indicating a type 1 T helper (Th1) cytokine–dominated immune response. Again, these mice not only develop significantly increased serum levels of IgG and IgA but also show AMAs specific for PDC-E2, typically found in human PBC [29].

Expression of AMAs, paired with immunological and pathological findings similar to human PBC, is also found in mice with a disrupted *Ae2*_*a,b*_ gene. Apart from an enlarged spleen, increased production of IL-12p70 and IFN-γ, an expanded CD8^+^ T-cell population and low numbers of CD4^+^FoxP3^+^/regulatory T cells, these mice show an extensive portal inflammation with infiltrating CD8^+^ and CD4^+^ T lymphocytes surrounding damaged bile ducts. Cholangiocytes isolated from these mice show gene expression changes compatible with oxidative stress and increased antigen presentation [30].

Another model for PBC are *NOD.c3c4* mice congenically derived from the nonobese diabetic strain that develop an autoimmune biliary disease resembling human PBC. These mice are completely protected from diabetes by B6/B10 regions on chromosomes 3 and 4 that contain B6/B10 insulin-dependent diabetes (*Idd*) loci. Furthermore, they develop AMAs to PDC-E2 that, as in human PBC, are specific for the inner lipoyl domain. Biliary duct inflammation shows infiltration with CD3^+^, CD4^+^ and CD8^+^ T cells. *NOD.c3c4* mice treated with monoclonal antibodies to CD3 are protected against biliary injury. In this model, the central role of T cells in developing characteristic symptoms of PBC can be shown. After performing an adoptive transfer of splenocytes or CD4^+^ T cells, *NOD.c3c4-scid* mice develop bile duct injury characterized by destructive cholangitis, granuloma formation and eosinophilic infiltration as seen in human PBC. However, *NOD.c3c4* mice also develop injury of the extrahepatic biliary ducts [31].

Bile duct injury is also inducible by immunization with different agents. Obviously, in most animal models mimicking human PBC, AMAs against PDC-E2 play a crucial role. Therefore, another mouse model was generated by the immunization of mice with 2-octynoic acid coupled to bovine serum albumin (2-OA-BSA), an antigen selected following quantitative structure–activity relationship analysis of PDC-E2. The immunization with and without the addition of α-galactosylceramide (α-GalCer), an invariant natural T-cell activator, leads to a profound exacerbation of autoimmune cholangitis, including significant increases in CD8^+^ T-cell infiltrates, portal inflammation, granuloma formation and bile duct damage [32]. This suggests a primary role of the innate immune system in the exacerbation of autoimmune cholangitis.

In addition to the above-mentioned models, several dietary models leading to cholestatic liver injury have been introduced. These agents include 3,5-diethoxycarbonyl-1,4-dihydrocollidine (DDC) or α-naphthylisothiocyanate (ANIT).

DDC feeding is widely used to study Mallory body formation (as seen in alcoholic liver disease) or oval cell activation and proliferation in murine models of liver injury. Moreover, cholestatic serum markers are significantly induced in these mice. Feeding mice a diet supplemented with 0.1% DDC for 8 weeks leads to increased biliary porphyrin secretion. A strong ductular reaction can be observed after one week. In epithelial biliary cells, the expression of cytokines such as vascular cell adhesion molecule, osteopontin and TNF-α is upregulated. Histopathologically, oral DDC uptake leads to pericholangitis with infiltration of inflammatory mononuclear cells and activation of periductal myofibroblasts, causing biliary liver fibrosis that resembles sclerosing cholangitis in humans [33].

Feeding mice ANIT is another xenobiotic model to induce cholestatic liver injury. In general, chronic biliary injury and increase in the number of bile canaliculi can be induced in mice by feeding them a diet supplemented with ANIT in low doses (0.025%), which results in cholestasis several days after feeding [34]. ANIT is conjugated with glutathione in hepatocytes and is transported into the bile by the Mrp2 transporter [35]. Because glutathione-conjugated ANIT is unstable in bile, it undergoes recycling rounds of absorption and metabolism, resulting in bile concentrations that cause direct biliary epithelial cell injury. This injury causes reactive expansion of the biliary epithelium, mild hepatocellular injury and periportal inflammation, which lead to biliary liver fibrosis [36]. Administration of a single large dose of ANIT (300 mg/kg body weight) to mice leads to rapid (15 to 24 hours) cholestasis induced by severe destruction of biliary epithelial cells and periportal hepatocellular necrosis [37]. Interestingly, similar intracellular signalling pathways are involved in the mediation of obstructive cholestatic injury (that is, BDL) and ANIT-induced injury. These pathways include the activation of TGF-β and α_V_β_6_ integrins [38,39].

All murine models of cholestatic liver fibrosis show several characteristics leading to liver injury: direct damage of the biliary epithelial cells induced by obstruction, autoimmune processes or xenobiotic-triggered immune responses leading to infiltration of mononuclear cells and periductular inflammation. Depending on the study aims, investigators should choose an injury model with characteristics most suitable for the study objective. For example, a BDL model can be used to study the effect of cholestatic injury in transgenic mice. Models with genetically induced biliary injury and strong autoimmune effects can give valuable information about inflammatory cell migration and recruitment. Therefore, in addition to carefully selecting the most suitable model for the study, the interpretation of overlapping effects of cell injury in those models is very important.

### Toxic models

Several well-established chemical substances have been identified that induce liver inflammation and fibrogenesis. The most commonly used approach to induce toxin-mediated experimental liver fibrosis is the periodic administration of carbon tetrachloride (CCl_4_) in mice or rats. In mice, typically 0.5 to 2 ml/kg body weight CCl_4_ (diluted in corn oil) is injected intraperitoneally (i.p.) two to three times per week, resulting in robust and highly reproducible liver fibrosis between 4 and 6 weeks of treatment. Long-term intoxication using inhalation is the standard method for the induction of cirrhosis with portal hypertension. Oral gavage is an alternative application route [40]. However, it was observed 40 years ago that oral CCl_4_ application is associated with frequent early mortality [41]. The susceptibility to CCl_4_-induced liver fibrosis in mice depends largely on genetic background. BALB/c inbred mice are most sensitive to fibrosis induction, whereas FVB/N mice respond significantly less to CCl_4_[42]. Although C57BL/6 inbred mice develop only intermediate liver fibrosis, this strain is frequently used for fibrosis studies in the CCl_4_ model because of the ready availability of respective knockout mutants or other gene modifications. CCl_4_ is metabolized by hepatocytes, giving rise to toxic trichloromethyl (CCl_3_) radicals, which mediate cytotoxic effects and eventually lead to massive centrilobular liver necrosis [43]. In addition, some evidence exists that CCl_4_ may induce apoptotic cell death of hepatocytes [44], although this might be a secondary effect and has not been investigated in more detail to date.

The kinetics of fibrosis development can be roughly divided into three phases: (1) acute injury, (2) initiation of fibre formation and (3) advanced fibrosis. The phase of acute CCl_4_-mediated liver fibrosis is characterized by activation of Kupffer cells and induction of an inflammatory response, resulting in secretion of cytokines, chemokines and other proinflammatory factors. This in turn attracts and activates monocytes, neutrophils and lymphocytes, which further contributes to liver necrosis [45] followed by a strong regenerative response that results in substantial proliferation of hepatocytes and nonparenchymal liver cells at around 48 hours after the first CCl_4_ application [46]. Thus, a single CCl_4_ injection in mice can also be used as an attractive and highly reproducible model of liver regeneration after toxic injury. The first appearance of histological fibrosis and scarring fibres is usually observed after 2 to 3 weeks of CCl_4_ treatment, depending on the dosage and mouse strains used. Molecular fibrosis markers are also easily detectable at this time. Accordingly, mouse mutants that are expected to display accelerated onset of liver fibrosis can be analysed after 2 weeks of continuous treatment. True bridging fibrosis can be observed after 4 to 6 weeks of continuous treatment, corresponding to approximately 8 to 18 injections. Of note, CCl_4_-induced liver fibrosis in mice can be completely resolved within several weeks after withdrawal of the toxic treatment [47,48]. Thus, the CCl_4_ model resembles all important properties of human liver fibrosis, including inflammation, regeneration, fibre formation and potentially fibrosis regression.

Likewise, continuous administration of thioacetamide (TAA) is another well-established model of experimental liver fibrosis in rodents. It was originally established in rats [49-51], but it is also frequently applied in mice and often serves as a second, independent approach to confirm data obtained from, for example, CCl_4_-treated animals. Although known as a potent inducer of liver injury for decades, the molecular mechanism of TAA-induced liver fibrosis is still not completely understood. TAA is bioactivated in the liver via oxidation processes leading to its *S*-oxide and the highly reactive *S,S*-dioxide, which is presumably responsible for TAA hepatotoxicity [52]. Earlier studies suggested that TAA bioactivation involves the hepatic cytochrome P450 enzyme CYP2E2 [53,54].

TAA can be administered i.p. at concentrations ranging from 150 to 200 mg/kg body weight three times per week [55,56] or given orally by adding 200 mg/L of TAA to the drinking water [57]. I.p. application of TAA results in hepatic centrolobular necrosis, elevated transaminase activity and robust liver fibrosis within 6 weeks. Interestingly, oral administration of TAA does not lead to significant elevation of transaminases in mice [57], thus contributing to a lower burden for experimental animals. In addition, this scenario closely resembles the situation in hepatitis patients with only mild elevation of aspartate aminotransferase (AST) and alanine aminotransferase (ALT), but it still has a high likelihood of leading to liver fibrosis. However, oral administration of TAA requires a much longer application to induce a similar strength of liver fibrosis in comparison to 6-week i.p. treatment with CCl_4_ or TAA. In addition, the impact of oral application of toxins on the gastrointestinal tract irritation that should be expected was not analysed in detail in these studies.

Although much less frequently used in fibrosis research, experimental liver fibrosis can also be induced by regular administration of the hepatocarcinogen dimethylnitrosamine (DMN) [58]. Its mode of function is very similar to that of diethylnitrosamine (DEN), which is described in detail further below. It has been described that i.p. injection of 10 mg/kg DMN twice weekly results in liver fibrosis within 4 weeks, which was associated with activation of hepatic stellate cells, Kupffer cells and expression of profibrotic cytokines [59], thereby defining DMN as a probate drug capable of inducing prototypical profibrotic mechanism. However, DMN also has strong mutagenic and carcinogenic properties. Therefore, the analysis of underlying profibrotic mechanisms in this experimental model could be more complex because of overlapping or even mutated signalling pathways.

Most studies still rely on the CCl_4_-model to induce toxic liver fibrosis in mice due to the good comparability with the abundance of previous publications, excellent reproducibility and moderate burden for the animals. When administrating TAA, the application mode should be carefully considered as i.p. application results in strong injury (similar to CCl_4_), while oral feeding mimics mild hepatitis reflecting e.g. alcoholic liver disease. The DMN model is especially attractive, if the progression from fibrosis to cancer is within the focus of interest.

### Animal models of metabolic liver injury

Nonalcoholic fatty liver disease (NAFLD) eventually leading to nonalcoholic steatohepatitis (NASH) is the most common chronic liver disease entity worldwide [60,61]. Although NAFLD describes the accumulation of simple fat inclusion in liver cells, NASH is characterized by an additional intralobular inflammation and hepatocellular ballooning. This eventually leads to fibrotic remodelling of the liver with the final risk of hepatocellular carcinoma (HCC) development. Pathogenetically, NASH can be considered the hepatic manifestation of the metabolic syndrome, which is defined by the appearance of central obesity, insulin resistance, glucose intolerance and dyslipidaemia [60,61].

**Figure 3:**
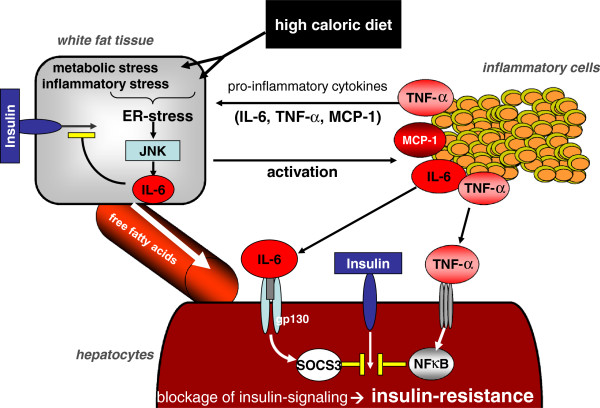
**Development of hepatic insulin resistance during nonalcoholic steatohepatitis.** Nonalcoholic steatohepatitis (NASH) pathogenesis and insulin resistance are based on the complex interplay between white fat tissue, hepatocytes and interfering inflammatory cells. A high-calorie diet induces metabolic and inflammatory stress in white fat tissue cells, which in turn releases free fatty acids in increasing amounts into the portal blood flow. In the liver, insulin resistance is then promoted through the release of proinflammatory cytokines provided by infiltrating inflammatory cells, which sustains the inflammatory response further. ER, endoplasmic reticulum; IL-6, interleukin 6; MCP-1, monocyte chemoattractant protein 1; NFκB, nuclear factor κB; SOCS3, suppressor of cytokine signalling 3; TNF-α, tumour necrosis factor α.

The development of fatty liver diseases is rather complex (Figure 3). Day and colleagues previously stated the 'two-hit’ hypothesis, which is considered the current model for NAFLD/NASH pathogenesis [62]. The first hit describes the development of steatosis in the liver based on an enhanced production rate of long-chain fatty acids, its impaired elimination due to impaired hepatic mitochondrial β-oxidation, as well as enhanced synthesis and secretion of triglycerides in hepatocytes. Furthermore, failure of synthesis of very low-density lipoprotein (VLDL) accounts for the development of steatosis. Steatotic livers are more sensitive to the induction of inflammation by a second pathogenic 'hit’. This postulated second hit could be oxidative stress, TNF-α signalling, apoptosis or mitochondrial dysfunction [63,64]. In cases where the capacity of mitochondrial oxidation is overwhelmed, alternative pathways in peroxisomes and the endoplasmatic reticulum obtain a more crucial role in hepatic fatty acid oxidation. Metabolites of these minor pathways then become sources of reactive oxygen species (ROS), which, as a result of the hepatic fatty-acid overload, leads to increased hepatocyte ROS content. In all cells and species, ROS overproduction then exceeds the antioxidant capacities (for example, by glutathione) of the cell, leading to nuclear and mitochondrial DNA damage, phospholipid membrane disruption by lipid peroxidation and eventually the release of proinflammatory cytokines [65,66]. ROS and metabolites of lipid peroxidation subsequently promote cell death due to damage of intracellular organelles and increased expression of the Fas ligand [65], which seems to be crucial for further NASH pathogenesis. However, the Fas receptor is usually sequestered by c-Met (the cellular receptor for hepatocyte growth factor with tyrosine kinase activity); thus Fas activation and the following induction of apoptosis is physiologically prevented. Interestingly, in cases of NASH, the Fas ligand is produced in excess and the inhibition through c-Met is restrained [67]. This in turn triggers cell death and inflammation while NASH progresses.

As a consequence of the accumulation of lipid peroxidation metabolites and ROS, as well as of the permanent inflammatory response involving multiple cytokines (TNF-α, IL-1β and IL-6) and an increase in TGF-β1 expression, HSCs become directly activated to produce scar-forming collagen, and therefore liver fibrogenesis develops. Additionally, free fatty acids induce the processing and activation of caspase 1 in Kupffer cells and hepatocytes, which promotes cleavage of IL-1 and therefore, ultimately, liver injury with a subsequent activation of HSCs. Further collagen accumulation then maintains the development of liver fibrosis, which can progress to cirrhosis and end-stage liver disease, including HCC development [68,69]. Although no animal model completely imitates the histology and pathophysiology of human NASH, several adequate genetic and dietary mouse models have been developed during the past few decades. Herein we focus on three different dietary models and one genetic model of NASH.

In the high-fat diet model of NASH, mice obtain 60% to 71% of their energy intake from an animal chow with special high-fat content, which is fed *ad libitum*. The results in this model may vary on the basis of the gender and genetic background of the animals. Feeding male mice a high-fat diet resulted in stronger hepatic lipid accumulation in Balb/C mice compared to C57BL/6J mice [70]. High-fat dietary experiments in rats revealed the development of steatohepatitis in Sprague-Dawley rats, but not in Wistar rats [71,72]. Administration of a high-fat diet results in enhanced plasma insulin levels, indicating the development of insulin resistance, which is an important attribute of the metabolic syndrome. Besides panlobular steatosis and strongly enhanced hepatic lipid content, increased transaminases and finally signs of hepatic inflammation and fibrosis were observed in rats after 4 weeks on a high-fat diet [71].

Almost 50 years ago, Lieber and DeCarli developed a liquid diet containing alcohol in a nutritionally adequate form for the study of alcohol-induced liver diseases [73]. However, this model induces only mild steatosis, slight elevation of transaminases and little or no inflammation in the absence of a secondary insult. Thus questions remained regarding whether it could truly serve as a model for the common problem of chronic alcohol intake and the subsequent development of liver diseases. Therefore, the protocol has been modified to better meet the needs of researchers interested in the investigation of dietary liver injury [74,75].

Feeding mice with a methionine and choline-deficient diet (MCD) leads to the development of steatosis and inflammation in the second week of treatment, which is clearly more rapid compared to the high-fat diet model [76,77]. The MCD diet contains 40% sucrose and 10% fat. Methionine and choline play a major role in the synthesis of phosphatidylcholine, which arranges the secretion of hepatic triglycerides [78,79] and the transport of VLDL out of the liver. With MCD chow, stearoyl-coenzyme A desaturase 1 (SCD-1), which is a key enzyme in the synthesis of triglycerides, is downregulated [80]. Oxidative stress, as determined by enhanced levels of enzymes of the P450 cytochrome system, in particular CYP2E1, and the improvement of steatohepatitis due to increasing antioxidant capacities, as well as alterations in cytokine and adipocytokine expression, also account for progressive liver injury [81,82]. Together with depletion of antioxidant factors such as glutathione, ROS promote oxidative stress and induce steatohepatitis and enhanced levels of TNF-α. An MCD diet induces stronger ROS production, mitochondrial DNA damage and apoptotic cell death compared to other dietary mouse models and is therefore one of the best-established model for NASH-associated inflammation. However, it also has some disadvantages. The amount of liver injury due to an MCD diet differs between mice and rats as well as between strains. A detailed comparative analysis of female 8- to 10-week-old mice from seven different inbred strains (A/J, AKR/J, Balb/cJ, C57BL/6J, DBA/2J, C3H/HeJ and 129X1/SvJwT), for example, revealed that the different mice showed an overall variation in regard to ALT, liver weight and liver fibrosis when fed an MCD diet [83]. Similar results were more recently reported in a study that compared chemokine (C-C motif) ligand 2 (Ccl2)-deficient mice on two different genetic backgrounds (that is, Balb/C and C57BL/6J) [84].

In addition, it is known that males develop stronger NASH attributes while showing less steatosis [85]. The most severe disadvantage is that the metabolic profile of the MCD model does not completely reflect all properties of NASH in humans. For instance, an MCD diet leads to particular weight loss of the animal in line with decreased plasma triglyceride and cholesterol levels. Besides serum insulin, leptin and glucose levels are reduced and adiponectin levels are unchanged or increased [81,86]. Strikingly little or no insulin resistance is present in this model [87].

The administration of a solely choline-deficient (CD) diet is an alternative for the induction of NASH. Choline, as described above, is important for degradation of VLDL and the secretion of triglycerides. A CD diet induces steatosis, inflammation and fibrosis over a period of 10 weeks. These mice exhibit no difference in body weight compared to the control group, which stands in clear contrast to mice fed an MCD diet [88]. In contrast, mice fed a CD diet were insulin-resistant and had higher plasma lipids compared to the MCD group, which, in contrast, had stronger steatosis and inflammation [89]. A CD diet supplemented with ethionine was then introduced as a model for stronger NASH development (referred to as the CDE model). The antimetabolite ethionine is a methionine antagonist and is usually provided in drinking water. However, this additionally hampers hepatocyte proliferation, making it a useful model for the study of hepatic progenitor cells [90].

Other alternatives for reproducing NASH in animals are genetically altered mouse models. One of the most widely used genetic NASH animal models is the *ob/ob* (ob = obese) mouse lacking functional leptin. Of note, leptin is an adipose tissue–derived hormone. These mice become extremely obese, hyperphagic, inactive and insulin-resistant, and they exhibit hyperglycaemia together with hyperinsulinemia and eventually develop hepatic steatosis [91]. Thus, within these mice, characteristic metabolic malfunctions clearly reflect NAFLD. However, this does not progress to steatohepatitis spontaneously. Additional stimuli such as an MCD or high-fat diet are therefore required [92,93]. Interestingly, these mice are resistant to fibrosis, even when treated with CCl_4_ or TAA, suggesting a crucial role of leptin in hepatic fibrogenesis [86,94,95].

Taken together, NASH development is the result of a complex sequence of metabolic, inflammatory and structural changes affecting liver physiology and function. Dietary models and genetic modified animals can be used to mimic changes appearing in human NAFLD and NASH, although none of these disease models completely reflects the disease development in its entire aspect. Therefore, the decision for or against a certain model should always be based on the particular aspect that is the focus of the study. This implies that different NASH models should be analysed in parallel to exclude experimental pitfalls.

### General aspects of liver fibrosis in animal models

#### ***Immunological mechanisms of fibrosis***

**Figure 4:**
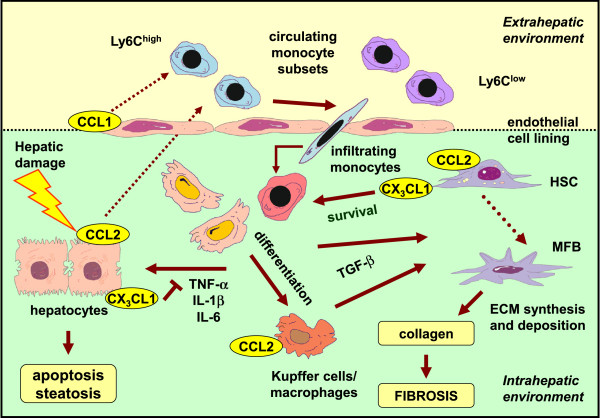
**Representative example of the complexity of the chemokine network regulating immune mechanisms during liver fibrosis.** Sophisticated experimental mouse models of chronic injury and fibrosis revealed the complex interplay of different hepatic cells and monocytes/macrophages during hepatofibrogenesis. Injury to the liver induces the expression and release of various chemokines (for example, chemokine (C-C motif) ligand 2 (CCL2), CCL1 and chemokine (C-X3-C motif) ligand 1 (CX_3_CL1)) from different hepatic cell subpopulations (for example, hepatocytes, sinusoidal endothelial cells, hepatic stellate cells (HSCs)). These chemokines potently chemoattract inflammatory Ly-6C-expressing monocytes from the circulation. As a consequence, these cells infiltrate the liver parenchyma, and monocytes differentiate into distinct macrophage subsets. Macrophages are a source of profibrogenic transforming growth factor β (TGF-β) that triggers transdifferentiation of HSCs into myofibroblasts (MFBs) responsible for excessive matrix formation and deposition (for example, collagen). On the other hand, macrophages also produce inflammatory cytokines (for example, tumour necrosis factor α (TNF-α), interleukin 1β (IL-1β) and IL-6) that altogether drive apoptosis and steatosis of parenchymal cells (that is, hepatocytes). ECM, extracellular matrix.

Inflammation is found in virtually all types of liver disease, and it has been recognized that persistent inflammation is the key driver of progressive liver disease, characterized by hepatitis, fibrogenesis, cirrhosis and hepatocellular carcinoma [96]. The immune reaction in the injured liver is a highly regulated process involving the activation of resident hepatic immune cells, such as Kupffer cells, massive infiltration of a variety of different immune effector cells, such as monocytes and lymphocytes, as well as direct and indirect interactions (for example, via cytokines or growth factors) of parenchymal and nonparenchymal liver cells with immune cells (Figure 4) [15]. In principle, two types of initiation of immune responses can be distinguished. In immune-initiated human liver diseases such as autoimmune hepatitis, some types of drug-induced injury and hepatitis B virus infection, activation of the immune system, including the adaptive part of immunity, directly promotes hepatotoxicity [97]. In all other cases, such as nonalcoholic or alcoholic steatohepatitis, classical drug hepatotoxicity or most cholestatic diseases, the injured liver (for example, necrotic or apoptotic hepatocytes) provokes the inflammatory reaction, largely involving innate immune mechanisms [96]. Of course, these initiation pathways are not mutually exclusive, and, at advanced disease stages, persistent injury and persistent inflammation are too closely linked to distinguish cause and consequence.

From an immunological point of view, the classical mouse models of liver injury mimic quite well the different immunological aspects of liver disease. For instance, immune-mediated initiation is responsible for liver damage upon Concanavalin A injection into mice [98], but also in new models for autoimmune hepatitis, in which hepatocyte-specific expression of antigens and antigen-directed T- and B-cell responses are achieved [99,100]. In contrast, classical murine or rat fibrosis models, such as CCl_4_ administration, surgical BDL and a steatohepatitis-inducing MCD diet, reliably provoke a defined inflammatory response within the injured liver [101].

A recent study reporting the lack of analogous gene array variations between human disease samples and mouse models of three major inflammatory conditions––sepsis, burns and trauma––has raised concerns regarding the reliability of mouse models in general for immunological research in defined disease models [102]. In fact, in studies using liver fibrosis models, several differences between murine and human immune cells in the liver need to be carefully considered, such as the different number and proportion of distinct immune cell populations in the liver between mice and humans and the different marker molecules to identify corresponding immune cell subsets between mice and humans [93]. For instance, the proportion of unconventional γδ T cells is lower in human liver than in mouse liver [103], the human neutrophil-attracting chemokine IL-8 has no direct analogue in mice (which employs CXCL1 to attract neutrophils) [104] and subsets of circulating classical and nonclassical monocytes show very different ratios in humans (90%:10%) and mice (50%:50%) [105]. Moreover, the genetic background of inbred mouse strains largely influences the responsiveness of their immune systems to specific stimuli (for example, rather Th1- or Th2-driven T-cell reactions), leading to different fibrogenic responses in standard mouse models of liver fibrosis, depending on the mouse strain [84,106].

Nevertheless, taking all these potential shortcomings into account, mouse models have been of outstanding value in detecting immunological reactions during hepatofibrogenesis. For instance, the strong increase of chemokine receptor CCR2 expression has been observed in human fibrotic liver samples for a very long time [107,108], but its functional relevance has remained obscure. Various mouse models of liver fibrosis conducted in independent laboratories revealed the CCR2-dependent accumulation of a distinct profibrogenic monocyte subset in acute and chronic liver injury [84,109-112]. The Ly-6C^+^ (Gr1^+^) monocytes in mice release proinflammatory (for example, TNF-α) and profibrogenic (for example, TGF-β) cytokines and can also directly activate collagen-producing stellate cells, thus representing a key mechanism for linking perpetuation of inflammation to development and progression of fibrosis [106]. This in turn prompted intense research in human fibrosis and led to the discovery of monocyte/macrophage subsets in human liver, assigning proinflammatory and profibrogenic functions to the subset of CD14^+^CD16^2+^ nonclassical or intermediate macrophages [113,114]. Therapeutic interventions based on these findings, such as inhibition of the chemokine CCL2 or transfer of beneficial macrophage subsets, are currently being evaluated in animal models as well as in early-phase clinical trials [109,115,116].

Another advantage of animal models is that they are useful for the study of cell–cell interactions in the context of the organ-specific microenvironment. For instance, it has been noted that *in vitro* activated, cultured HSCs largely differ in their gene array profiles from *in vivo* activated HSCs [117]. This discrepancy was reduced when HSCs were cocultured with hepatic macrophages [117], prompting subsequent *in vivo* studies in mice that revealed an intimate cross-talk between HSCs and macrophages [118]. This principal finding was later confirmed in primary human HSCs and macrophages and was even assigned to distinct cellular subsets [113,115].

Besides a central role of monocytes/macrophages as key initiators and perpetuators in the progression of liver fibrosis, the liver (both mouse and human) is highly enriched by unconventional lymphocytes, including natural killer (NK) cells, NK T (NKT) cells and γδ T-cell receptor–expressing T cells. In conditions of chronic liver injury, T cells also represent a major lymphocyte component of the inflammatory infiltrate [15]. In many cases, human studies have described the presence and allowed phenotypic characterization of the different cell types, whereas mouse models have been invaluable in defining the functional contribution of these cells. For instance, NK cells are capable of promoting HSC apoptosis and are thus considered antifibrotic in murine and human fibrosis [119,120]. CD8 T cells, on the other hand, induce liver fibrosis by activating HSCs [121], and CCR7 has been associated with infiltration of CD8 T cells [122]. The chemokine receptors CCR5 and CXCR3 have been described as being involved in CD4 T-cell recruitment to the liver in mice and humans [123-126]. Among the CD4 T-cell populations, IL-17–expressing Th17 cells have gained much interest in fibrosis research because they are thought to exert important proinflammatory and profibrogenic actions in humans and mice [127-129].

Taken together, acute or chronic injury to the liver provokes the highly regulated and controlled activation of distinct immune cells from the innate and adaptive immunity, which critically initiate and perpetuate inflammation and promote fibrogenesis. The thorough dissection of immune cell–related functions from animal models has provided profound insights into the pathogenesis of liver fibrosis, and translational studies have confirmed the relevance of findings derived from mouse and rat models for human liver diseases. The tremendous advances in deciphering immunological mechanisms in liver fibrosis in mouse models and human samples gives rise to the expectation that these pathways will translate into novel therapeutic approaches for hepatic fibrosis in the near future.

#### ***Targeting specific cells involved in fibrogenesis***

As outlined above, liver fibrogenesis involves activation and interaction of several hepatic cell types upon the chronic injury of which the most prominent are HSCs, hepatocytes, Kupffer cells and monocytes. Thus, targeted manipulation of each of these cell types could be of great benefit for the treatment of liver fibrosis. In addition, cell type–specific deletion or overexpression of pro- and antifibrotic genes is still a major goal of basic fibrosis research. This aim has been facilitated by the implementation of the *Cre*/*loxP* recombination system in mice and the characterization of powerful cell type–specific promoters driving *Cre*-mediated gene deletion exclusively in the target cell of interest [130]. Regarding the liver, most advances have been made by deleting target genes in hepatocytes. Transgenic expression of Cre recombinase under the control of the albumin promoter/α-fetoprotein enhancer (*Alfp*-*Cre*) is well-established and allows deletion of *loxP*-flanked target genes in hepatocytes with efficiencies of 95% and higher [131,132].

Targeting HSCs is presumably more relevant for development of therapeutic approaches, as these cells are the major source of ECM in the liver, especially during fibrogenesis [6]. Therefore, current approaches aim to express *Cre* recombinase specifically in HSCs. Several recent reports have demonstrated that the promoter of glial fibrillary acidic protein (GFAP), which is activated in resting HSCs and astrocytes, is able to drive *Cre*-mediated target gene deletion (*GFAP*-*Cre*) in HSCs, but not in other hepatic cell types. This approach was successfully used to track hepatic stellate cells *in vivo* by *Cre*-mediated reporter gene activation (for example, enhanced green fluorescent protein, *EGFP*) under control of the *GFAP* promoter [22]. In other recent studies, *GFAP-Cre*–transgenic mice were successfully used to study the role of autophagy and senescence in HSCs during fibrosis progression. *GFAP-Cre*–driven deletion of autophagy-related protein 7 (*ATG7*) in hepatic stellate cells in mice following CCl_4_ or TAA treatment reduced matrix accumulation and liver fibrogenesis [133]. To investigate the role of HSC senescence for fibrosis progression, the tumour suppressor *p53* was selectively deleted in HSCs using *GFAP-Cre* mice, which prevented cellular senescence, enhanced liver fibrogenesis and unexpectedly triggered non–cell autonomous tumour-promoting mechanisms in macrophages [134].

Additional strategies have been developed to induce *Cre* expression specifically in activated, collagen-producing HSCs, even in an inducible manner. A very recent study described the sophisticated generation of a *Cre* transgene in mice, which was fused to a mutant oestrogen ligand-binding domain and controlled by the murine vimentin promoter [135]. As a consequence, *Cre* expression in the respective mice requires the presence of tamoxifen (an oestrogen receptor antagonist) and also of vimentin, which is predominantly expressed in myofibroblast-like cells such as activated HSCs [135]. Accordingly, administration of tamoxifen at a desired time point allows *Cre*-mediated deletion of target genes or activation of reporter genes specifically in activated stellate cells. However, the potential weaknesses and virtues of this strategy have to be evaluated in future studies.

Kupffer cells and monocytes are important for the progression of liver inflammation and fibrosis [136]. Although Kupffer cells represent the population of resident macrophages within the liver, monocytes are recruited to the liver upon specific trigger and can be considered the circulating precursors of tissue macrophages and dendritic cells. Genetic targeting of profibrotic genes in these two cell populations could be of high value for understanding cellular cross-talk during liver fibrosis. Surprisingly, very few studies to date have aimed to target genes specifically in Kupffer cells/monocytes in experimental liver fibrosis. The generation of mice expressing *Cre* in the myeloid lineage under control of the murine M lysozyme locus was described more than one decade ago [137]. In these mice, *Cre* recombinase is expressed in monocytes, macrophages and neutrophils, but with some variation. However, few studies have used this approach for analysis in experimental fibrosis [138,139]. Similarly, transgenic mice with *Cre* expression in resident macrophages under control of the F4/80 promoter were described in a 2002 study [140]. Of note, the F4/80 molecule is a cell surface glycoprotein expressed at high levels on the surface of several resident macrophages, including Kupffer cells in the liver [140], but only one study published to date [139] has described the use of this strain for a liver-specific analysis.

Several tools and transgenic mice are available for cell type–specific targeting of fibrosis-relevant cells. Targeting includes genetic labelling of cell types or cell type–specific deletion of pro- and antifibrotic genes. Although tools for targeting hepatocytes and HSCs are well-developed and have been improved, the literature on genetic targeting of monocytes/macrophages in the fibrogenic liver is still limited, presumably due to a lack of efficient, cell-type–specific, *Cre*-transgenic mice.

Drug-targeting and the development of specific delivery systems to the liver have recently become a very important focus in fibrosis research. At present, no effective pharmacological intervention is available to treat human liver fibrosis. Although current advances in genetic targeting of specific cell populations have greatly contributed to the identification of genes, cells and mechanisms involved in liver fibrogenesis, these strategies are barely applicable for human therapy, but they do help to define suitable therapeutic targets. It has been widely accepted that HSCs play a critical role in liver fibrogenesis, as they are the main source of fibrotic ECM. Thus, drug-mediated targeting of profibrotic factors in HSCs is a major goal of current research, as reviewed in detail recently [141-143]. Herein two examples of promising drug-targeting strategies in HSCs will be introduced in more detail.

Activated HSCs show increased expression of the mannose 6-phosphate/insulin-like growth factor II receptor (*M6P/IGF2R*). It was previously shown that human serum albumin modified with mannose 6-phosphate specifically binds to *M6P/IGF2R* on activated but not on quiescent HSCs and gets effectively internalized, suggesting that mannose 6-phosphate substituted proteins are promising HSC-selective carriers for antifibrotic drugs [144]. This strategy was recently applied in a translational approach in rats [145]. Rho kinase is involved in enhanced portal pressure during liver cirrhosis. Using a Rho kinase inhibitor coupled to a mannose 6-phosphate/human serum albumin carrier, fibrosis progression, and especially portal pressure, could be substantially reduced without major systemic effects. Another promising approach took advantage of strong expression of the platelet-derived growth factor β receptor (PDGFβR) in activated HSCs [146]. In this study, IFN-γ, a cytokine with proven antifibrotic properties, was conjugated to a PDGFβR-specific carrier and administered to human HSCs and CCl_4_-treated mice [147]. In cells, conjugated IFN-γ showed PDGFβR-specific binding and full bioactivity, whereas drug delivery to mice revealed inhibition of profibrotic genes and reduction of hepatic fibrogenesis.

Current advances in HSC-specific drug delivery are promising. However, comprehensive further analyses in animal models will be necessary to identify the best-suited drug target and most optimal delivery strategies with minimal side effects before studies in patients with liver fibrosis are feasible.

### Complications of fibrosis in animal models

#### ***Portal hypertension***

Portal hypertension is one major complication occurring in human liver disease and in animal models of fibrosis. *Portal hypertension* is defined as the gradient between the portal pressure and hepatic venous (or central venous) pressure above 5 mmHg, as well as in human and animal models [148,149]. The main reason is a pathologically elevated intrahepatic resistance to portal blood flow due to fibrosis or cirrhosis caused by different chronic, mainly inflammatory, stimuli [148,150]. The site of the increased resistance may be prehepatic (portal vein obstruction) or posthepatic (hepatic vein obstruction). These mainly vascular forms are not within the scope of this review and have been discussed elsewhere [151,152]. There are two steps that are decisive and have the potential for cure: early interruption of liver damage and liver transplantation [153,154].

Otherwise, different noncurative strategies are available for the treatment of chronic liver disease. Most of these target portal hypertension [154]. By contrast, interruption or regression of fibrosis is much more difficult to achieve [153]. Therefore, research in this field is urgently needed, requiring appropriate animal models.

To develop new treatment strategies, understanding of the involved pathophysiological phenomena is pivotal. On the one hand, hepatic resistance is increased due to mechanical obstruction within the sinusoidal flow caused by fibrosis derived from inflammation and/or hepatocellular injury, and, on the other hand, contraction of myofibroblastic cells (portal myofibroblasts and activated HSCs) and smooth muscle cells contributes actively to intrahepatic obstruction [150,155]. The resulting portal pressure increase is associated with vasodilatation in the splanchnic bed and consecutive splanchnic hyperperfusion [156]. Besides this vasodilation, neoangiogenesis takes place, supporting formation of collaterals and shunts [157]. In parallel, a hyperdynamic circulation occurs with increased cardiac output, a situation seen quite consistently in humans, mice and rats with liver cirrhosis and portal hypertension [156]. Secondary to this, renal perfusion is often compromised, which results in sodium retention and ascites formation. Most of these pathogenetic features can be found in preclinical animal models of portal hypertension [148,158-160].

Animal models used to study portal hypertension and liver cirrhosis mainly comprise rats, rabbits and, less often, mice. The main reason is that haemodynamic measurements, for example, of portal pressure or systemic circulatory parameters, are easier in these larger animals, with higher reproducibility and reduced latency. However, mice offer the opportunity for genetic modification and therefore are indispensable for future research in portal hypertension, despite the drawbacks described elsewhere [151,152,155].

As outlined above, widely applied models for the induction of liver injury include bile duct ligation, CCl_4_ intoxication and application of TAA or DMN. In this section, we selectively refer to the specific characteristics of these treatments regarding portal hypertension during progressive fibrosis.

The BDL model induces mainly cholangiocyte proliferation with consecutive formation of peribiliary plexus and portal fibrosis, leading to portal hypertension and shunts within four to six weeks [161-163]. The animals show clear signs of portal hypertension with ascites, splenomegaly and splanchnic and systemic vasodilation, which are associated with decreased arterial pressure as well as intra- and extrahepatic angiogenesis [161]. In contrast to humans, the renal perfusion in BDL rats is increased, despite decreased creatinine and sodium excretion [158,159]. The main advantages of this model are technical feasibility, short time to achieve typical disease, reproducibility and high similarity with humans in terms of portal hypertension. One of the drawbacks in rats and mice is the development of a biliary cyst compressing the portal vein and the stomach. Setting the ligation far within the hilum or injecting Ethibloc or formalin into the bile duct prior ligation prevents this problem [164-166].

The CCl_4_ model of liver injury is used in mice, rabbits and rats and leads to cirrhosis with portal hypertension [40,149,151,167-169]. In rats, the routine technique to achieve portal hypertension is inhalation using different protocols until ascites is present as an unequivocal sign of portal hypertension. One study investigated different protocols of CCl_4_ administration in mice (subcutaneous, i.p. and different protocols of inhalation), showing the best results with regard to mortality and degree of portal hypertension in short-cycle, thrice-weekly inhalation [40]. As an unwanted complication, all the inhalation groups developed significantly more ascites than those receiving CCl_4_ subcutaneously and i.p. [40]. Compared to the BDL model, the CCl_4_ model shows portal hypertension of similar dimension, whereas systemic haemodynamic alterations are more moderate [149,170]. Of note, generation of ascites and related portal hypertension takes approximately 12 to 16 weeks of treatment, and cirrhosis together with ascites rapidly regresses within 7 to 10 days after withdrawal of CCl_4_[40,169,171].

Portal hypertension is also a consequence of TAA treatment in experimental models performed in rats and mice [172,173]. The toxin affects both the perivenular and periportal areas. The induction of cirrhosis with severe portal hypertension using TAA usually takes longer than CCl_4_ application (14 to 20 weeks), with lower incidence of ascites [173,174]. Once TAA-induced cirrhosis is established, it remains stable for several weeks even if TAA is withdrawn, which is a major advantage of this model. A weakness of this model, apart from the time-consuming procedure, is the fact that animals develop cholangiocarcinoma around 18 weeks after TAA intoxication [175].

DMN administration induces centrilobular hepatocellular necrosis. The chronic intoxication with DMN, usually as an i.p. application, leads to cirrhosis with ascites (the most reliable sign for established portal hypertension) in rats after around 13 weeks [58,176]. The drawback of this model is its high carcinogenic potential for animals; therefore, in our view, it is not a preferable model for fibrosis with portal hypertension.

NAFLD might progress to end-stage liver disease and portal hypertension as well [176,177]. Specific diets (for example, MCD or low protein and choline and enriched with fat) are used to induce liver steatosis and fibrosis in rats (see discussion above). After feeding for 12 to 24 weeks, these animals may develop cirrhosis with portal hypertension. These models are rarely used for induction of portal hypertension because the haemodynamics of these animals has not been properly characterized to date.

### Animal models of hepatocellular carcinoma

HCC represents the most common primary carcinoma of the liver [178]. It arises almost exclusively in a setting of chronic inflammation and subsequent liver fibrosis caused by a variety of pathogenic entities, such as viral hepatitis, fatty liver disease, chronic alcohol consumption and others [179]. The worldwide spread of viral hepatitis in the past, and in increasing numbers of patients with metabolic liver disease in Western industrialized countries, has resulted in a steep rise in HCC incidence in recent decades. Consequently, HCC is the third-leading cause of cancer-related death worldwide [180]. At present, therapeutic options against HCC are still limited. Although the multikinase inhibitor sorafenib (Nexavar; Onyx Pharmaceuticals, South San Francisco, CA, USA) represented the first systemic treatment with a significant survival benefit for HCC patients in a palliative setting [181], further large clinical trials evaluating new drugs with molecular targets similar to those of sorafenib recently failed [182], illustrating the urgent need for the evaluation of novel molecular targets to prevent and treat HCC.

To gain better functional insight into the molecular mechanisms of hepatocarcinogenesis, multiple studies were performed using human HCC tissue. On the basis of these studies, a collection of genetic and epigenetic alterations, chromosomal aberrations, gene mutations and altered molecular pathways were described [183]. However, in many cases, it was difficult to assess whether these alterations represented a correlative epiphenomenon or if they were causally linked to HCC pathogenesis. In the light of these aspects, animal models of HCC offer a unique possibility to study mechanistic and cellular aspects of tumour biology, including the genetics of tumour initiation and promotion, tumour progression and metastasis *in vivo*. Moreover, animal models also represent a valuable tool with which to prescreen various therapeutic compounds for their efficacy to inhibit particular signalling pathways and thus to prevent or decelerate HCC development and growth [184].

There are numerous mouse models available for HCC research, which can be broadly divided into (1) xenograft models, (2) chemically induced models and (3) genetically modified mouse models [45]. Whereas, tumours are formed by injecting human cancer cells into immune-deficient mice in xenograft models, HCC in chemically induced and genetic models arise in their natural cellular and intercellular context, allowing researchers to study molecular mechanisms and cellular interactions during tumour initiation. Thus these models are used much more frequently today. Therefore, exemplary chemically induced and genetic models are briefly introduced and discussed next.

DEN is most often used as a carcinogenic agent to induce cancer in the liver and is frequently applied as a single-dose i.p. injection [184]. The carcinogenic effect is due to its capability of alkylating DNA structures, comprising a two-step bioactivation process [185]. Initially, the DEN model was often used as a two-stage model in which initiation by DEN was followed by a promotion phase, with phenobarbital used as a promoting agent [186]. However, depending on the dose and time point of injection, a single injection of DEN can induce HCC after a period of latency. As such, injection of DEN at a dose of 25 mg/kg at day 18 into C57BL/6 mice results in liver tumour development at the age of 8 months. For the initiation-promotion model, 4-week-old mice are typically injected i.p. with 100 mg/kg DEN and, after an additional 4 weeks, receive 0.07% phenobarbital in drinking water, resulting in HCC development after 6 months.

Genetically, the DEN model resembles human HCC associated with a poor prognosis [187]. As stated above, it is the most widely used tumour model and has several advantages. (1) It can be easily administered to mice from different genotypes. (2) It has a high HCC incidence. (3) It is highly reproducible [45,188,189]. It has been widely used to study the role of inflammatory and stress-related signalling pathways in the initiation and promotion of liver cancer [190-192]. Importantly, this model is extremely well-tolerated by mice and is not associated with serious side effects. DEN treatment itself is not linked with an impact on survival within the first 12 months after treatment [193], showing that the hepatic tumour burden induced by this treatment does not affect overall liver function as is true in most other primary liver tumour models in mice. Recently, the protocol for DEN administration was optimized by the group of Schwabe: HCCs were induced in C3H/HeOuJ and C3H/HeJ mice by i.p. injection of DEN (100 mg/kg) at ages 6 to 14 weeks, followed by 6 to 12 biweekly injections of CCl_4_ (0.5 ml/kg i.p. dissolved in corn oil) [194]. By this modification, the authors demonstrated that the processes occur in the natural course of human liver disease––chronic hepatitis leading to liver fibrosis as the basis of liver cancer–could be even more closely mimicked. Again, the authors did not report any increased mortality of mice within the observation period or an increased rate of peritoneal or other infections [194].

In contrast to the DEN model, the *Mdr2*-knockout mouse (*Mdr2*^-/-^) represents a bona fide genetic liver tumour model. These animals lack a biliary transporter protein denoted as multidrug resistance gene 2 (*mdr2*), which prevents spontaneous cholestatic hepatitis and liver cancer [27]. Tumour development in these mice progresses through distinct phases: inflammation, hepatic fibrosis, dysplasia, dysplastic nodules and carcinomas, thus mimicking the formation of HCC in humans [195]. Recently, other genetic tumour models with similar sequences of disease progression have been described, such as conditional liver-cell–specific knockout mice of the TNF-dependent signalling genes *Nemo* and *Tak1*[196,197] or mice with hepatocyte-specific overexpression of the proinflammatory cytokine lymphotoxin [198]. All these tumour models have common features. As such, HCCs are mainly characterised by their histological features and rarely metastasize [184]. Moreover, genetic profiling and functional studies have revealed similar transcriptional profiles and molecular behaviour with regard to proinflammatory signalling pathways, as seen in human liver cancer [191]. None of these respective studies described a significant influence of the hepatic tumour load on liver functional parameters or the behaviour of mice compared to their littermates that did not carry the carcinogenic mutation [195,196,198].

Taken together, murine HCC models offer the unique chance to study the role of intracellular molecular pathways and immunological processes in the critical steps leading from chronic hepatic inflammation to liver fibrosis and liver cancer. These models have been newly characterized and optimized in recent years to better mimic the typical disease sequence seen in human patients with chronic liver disease. Moreover, these models are very well-tolerated and barely limit life expectancy or change the behaviour of mice during the typical observation periods. On the basis of the currently limited treatment options for liver cancer, these models are essential to identify novel targets for future drug-targeting approaches that might help to reduce the global challenges associated with this disease.

### Considerations and perspectives

#### ***Ethical and legal issues in performing animal experimentation***

During the past few decades, the public’s consciousness regarding animal welfare, especially in Europe, has dramatically changed. As a consequence of the debates surrounding this issue, within the year 2013, all member states of the European Union (EU) have to incorporate into their national laws the criteria of EU-Directive 2010/63 on the protection of animals used for scientific purposes.

This directive contains 66 articles and lays down rules for protection of nonhuman primates, animals taken from the wild, stray and feral animals of domestic species and animals bred for use for invasive or noninvasive animal experimentation or other scientific purposes (that is, so-called procedures) [199]. This implementation will foster the status of the 3R principle (reduction, refinement and reduction) set forth by Russel and Burch [18]. As a consequence, this law will enforce the 3R principle in animal experiments.

In particular, member states of the EU shall ensure that, wherever possible, a scientifically satisfactory method or testing strategy not entailing the use of live animals shall be used instead of a procedure [199]. Moreover, the directive requires that all procedures are classified in the future as 'nonrecovery’ , 'mild’ , 'moderate’ or 'severe’ and that all personnel who carry out experiments on animals have an adequate education. There is no doubt that these new laws will protect animals. It is also obvious, however, that there are no strict classification criteria for pain. Small variations in animal housing, anaesthesia, setting of experimental damage (for example, BDL) might further affect animal discomfort. In addition, time required for documentation, training of personnel and state monitoring will increase enormously. Moreover, because each laboratory has potentially susceptible and nonsusceptible variations in experimental protocols, it is obvious that strict guidelines and standard operating procedures for each disease model are required.

Within our Collaborative Research Centre SFB TRR57, Organ Fibrosis: From Mechanisms of Injury to Modulation of Disease, different models for the induction of organ fibrosis in rodents (mice and rats) are used in a highly standardized manner (for details about the aim of the SFB TRR57, see http://www.sfbtrr57.rwth-aachen.de/). To investigate different molecular mechanism and pathways involved in fibrogenesis, we apply methods in which the pathophysiology is induced via special diets (for example, CD and MCD), cholestasis (BDL), application of toxins (CCl_4_, DMN and DEN) or genetic modification of embryonic stem cells and the development of genetically modified organisms. In our consortium, we have strict, highly standardized protocols for each procedure. However, we are still aware of the fact that these standard operating procedures may potentially have to be adjusted to other consortia and the international comparability reviewed.

For this purpose and to foster meta-analysis according to standards comparable to evidence-based medicine, the Animals in Research: Reporting In Vivo Experiments (ARRIVE) guidelines that were originally published in 2010 [200] are, in our view, most suitable.

The aim of these consensus guidelines is to report animal studies in a systematic form to improve the ability for later analysis and to improve the reproducibility of experiments. Therefore, the ARRIVE guidelines are a useful refinement tool, but the scientific community has to foster refinement and reduction on its own. This can be done by avoiding unnecessary variations and applying state-of-the-art knowledge. The outcome of a particular diet is, for example, dependent on the genetic background of the mice [84] as well as on the different time intervals when the diet is used [201].

Most animal welfare standards in the new EU directive are similar to those prevailing in the United States. However, several of the new EU directive legal requirements exceed U.S. practice. It would be a great advantage if all details of animal experimentation were reported rigorously in related scientific publications. Referees of peer-reviewed journals should be aware of the fact that the request for additional animal experimentation will need additional approval by legal institutions and therefore will be more time-intensive in the future.

Another ethically important consideration mandated by the EU directive is the consideration and implementation of humane endpoints. No experiment should have as the only readout the death of the animal without any intervention, analgesia or the definition of a human endpoint termination of the experiment. Again, different diets also can have an impact ranging from moderate to severe lasting harm, and a generally accepted objective humane endpoint that were previously widely used in various experimental animal models is loss in body weight [202-206]. With this objective parameter, it is possible to define a scientific and human endpoint that is clear, measurable and objective [204]. However, in most of the experimental procedures, more than one human endpoint should be used to have a solid decision basis, and score sheets for each animals subjected to experimentation are useful to monitor the health-related status of the individual animals and the criteria for when to stop the experiment to avoid unnecessary pain, distress or harm. Moreover, the burgeoning use of genetically modified animals that we and others have used in our experiments might be another challenging task in establishing new forms of harm reduction through improved genetic modification technologies, plus continued attention to alternative approaches and cost–benefit analyses that include the large numbers of animals involved indirectly (for more critical discussion, see [207]).

The new animal welfare rules will change liver fibrosis research, at least in Europe. This change will include the establishment of highly standardized standard operating procedures, thereby increasing the reproducibility and comparability of results obtained from different laboratories.

### Translation of experimental findings from rodents to patients

As outlined above, animal models are still the gold standard for basic liver fibrosis research, especially due to the complex interaction of several cell types (hepatocytes, immune cells and HSCs) during fibrogenesis, which is challenging to mimic *in vitro*. As a consequence, various surgical, genetic, toxic and nutritional models are widely applied and serve as models for the different types of fibrosis observed in humans (Table 1). However, a very recent study has generally questioned the benefit of rodent models for inflammatory research [102]. In that report, the authors compared the gene expression changes in blood cells between mice and humans in three different inflammatory scenarios (burn, trauma and sepsis). Surprisingly, the expression profiles between mouse models and human disease revealed poor correlation. Although the affected primary organs (that is, skin and liver) have not been investigated, the authors of that study concluded that mouse models poorly reflect human inflammatory disease. In the context of that provocative study, liver fibrosis researchers have to carefully reevaluate if animal models of liver fibrosis are indeed appropriate approaches for understanding and healing human liver disease.

**Table 1:** **Overview of mouse models of liver fibrosis**^**a**^

**Animal model**	**Intervention**	**Advantage**	**Disadvantage**	**Type of fibrosis**	**Reference**
Bile duct ligation (BDL)	Surgical	Fast and highly reproducible		Cholestatic fibrosis	[22]
*Mdr2*^-/-^ mice	Genetic	Well-reproducible	Long latency (3 to 6 months)	Sclerosing cholangitis/biliary fibrosis	[27]
Dominant-negative *TGFβRII* mice	Genetic	Resembles human disease		Primary biliary cirrhosis (PBC)	[28]
*IL-2Ra*^-/-^ mice	Genetic	Resembles human disease		PBC	[29]
*NOD.c3c4* mice	Genetic	Resembles human disease	Injury of the extrahepatic biliary ducts	PBC	[31]
3,5-Diethoxy-carbonyl-1,4-dihydrocollidine (DDC)	Feeding	Resembles human disease		Sclerosing cholangitis with oval cell activation	[33]
α-Naphthylisothiocyanate (ANIT)	Feeding	Fast		Cholestatic fibrosis	[34]
CCl_4_ treatment	Injection, oral	Highly reproducible, fast, resembles properties of human fibrosis, good comparability due to abundant reference studies	Enhanced mortality by oral application	Toxic fibrosis	[43]
Thioacetamide (TAA) treatment	Injection, feeding	Injection, fast	Feeding, long latency	Toxic fibrosis and hepatocellular carcinoma (HCC)	[49-51]
Dimethylnitrosamine (DMN)	Injection	Fast	Mutagenic and carcinogenic	Toxic fibrosis and HCC	[58]
High-fat diet	Feeding	Fast, resembles features of insulin resistance and metabolic syndrome		Steatohepatitis and subsequent fibrosis	[70,71]
Lieber-DeCarli diet	Feeding	Well-tolerated	Long latency, only mild injury	Alcohol-induced liver fibrosis	[73]
Methionine- and choline-deficient (MCD) diet	Feeding	Fast, strong steatohepatitis along with elevated TNF	Metabolic profile only partially reflects human NASH, no insulin resistance, body weight loss, different outcome in different mouse strains	NASH-associated fibrosis	[76,77,83,84]
CD (solely choline-deficient) diet	Feeding	Resembles sequence steatosis -inflammation - fibrosis		NASH-associated fibrosis	[88]
Choline-deficient, ethionine-supplemented (CDE) diet	Feeding	Stronger NASH development compared to CD, activates hepatic progenitor cells		NASH-associated fibrosis	[90]
*ob/ob* mice	Genetic		Does not progress spontaneously to NASH or fibrosis	Fatty liver disease	[91]
Diethylnitrosamine (DEN) treatment	Injection	High HCC incidence, highly reproducible, well-tolerated, not associated with serious side effects	No development of fibrosis	Resembles human HCC associated with poor prognosis	[184]
DEN/CCl_4_ treatment	Injection	Reflects all stages of human liver disease from chronic hepatitis leading to liver fibrosis		Resembles naturally occurring HCC progression	[194]
Liver cell–specific *Nemo*^-/-^ mice	Genetic	Spontaneous fibrosis development		Cholestatic fibrosis and HCC	[196]
Liver cell–specific *Tak1*^-/-^ mice	Genetic	Spontaneous fibrosis development		Cholestatic fibrosis and HCC	[197]

^a^CCl_4_, Carbon tetrachloride; NASH, Nonalcoholic steatohepatitis; TNF, Tumour necrosis factor.

Within our consortium, Organ Fibrosis: From Mechanisms of Injury to Modulation of Disease, we have successfully shown that the animal models outlined above are extremely helpful to understand general pathophysiological pathways of fibrogenesis in humans. For example, patients with chronic liver disease show elevated expression of the chemokine CXCL16 and its cognate receptor CXCR6. Likewise, *Cxcl16* is strongly expressed by endothelium and macrophages in mice, whereas murine lymphocyte populations (NKT, NK, CD4 T and CD8 T cells) express CXCR6. Animal models of fibrosis (CCl_4_, MCD feeding) in combination with genetic knockout approaches targeting CXCR6 enabled us to unravel the underlying mechanism showing that hepatic NKT cells provide CXCR6-dependent signals early upon injury, thereby accentuating the inflammatory response in the liver and promoting hepatic fibrogenesis. Interfering with CXCR6/CXCL16 might therefore bear therapeutic potential in liver fibrosis [208].

One of the most established animal models of cholestatic liver disease comprises genetic deletion of the *Mdr2* gene in mice [27]. There are several independent reports that have shown that mutations or polymorphisms in the human *MDR2* homologue are associated with different entities of cholestatic liver disease in patients [26,209,210], which in turn underlines the benefit of animal models for clinical fibrosis research.

In another example, our studies in human biopsies from patients with liver fibrosis revealed increased cell-cycle activity of hepatocytes and HSCs that was associated with elevated expression of cyclin E1 and the proto-oncogene c-*myc*[211,212]. The use of appropriate transgenic mouse models confirmed these findings in rodents and eventually clarified the underlying mechanism defining cyclin E1 as a promising new therapeutic target.

Recent work by our consortium has shown that animal models allow identification of novel biomarkers suitable to detect inflammatory and fibrotic liver disorders in both animals and humans. Such a biomarker is lipocalin 2 (LCN2), which belongs to the superfamily of lipocalins representing a group of small secreted transport proteins. We have demonstrated that LCN2 is strongly increased in experimental models of acute liver injury and that animals lacking LCN2 are more prone to hepatic fibrogenesis [213,214]. On the basis of these findings, we suggested that LCN2 plays a pivotal role in liver homeostasis. In line with this hypothesis, we could demonstrate that LCN2 is a reliable indicator of liver damage in patients with diseased livers [213].

Portal hypertension and ascites are key complications of liver cirrhosis which are found in humans (regardless of aetiology) as well as in all animal models of liver cirrhosis [156,157,215]. Interestingly, vascular hypocontractility of the splanchnic and systemic arterial bed always occurs in rodents and humans. Furthermore, the initiators and pathways leading to an overproduction of the vasodilative nitric oxide are quite similar and accompanied by highly increased expression of vasoconstrictors [156]. Because of the similarity of all these factors, animal models are quite appropriate to study the pathophysiology of portal hypertension, its consequences and treatment options. Consistently, portal pressure and HSC activation in the BDL model was reduced after oral application of statins by influencing intracellular signalling [160,216]. Similarly, it has been shown that, in fact, statins also reduce portal pressure in humans and improve liver function [151]. Portal hypertension is associated with activation of the renin-angiotensin system, which involves binding of angiotensins to the G protein–coupled receptor Mas (MasR) [217] in an attempt to maintain systemic vascular filling and blood pressure. A recent publication demonstrated that the angiotensin–MasR axis controls similar or even identical signals in both cirrhotic rats and patients [218], again underpinning the power of animal experimentation.

Moreover, liver samples obtained from various disease models have been used to develop novel quantitative biometal imaging techniques allowing quantification of various metals in healthy and fibrotic and/or cirrhotic human liver specimens [219,220]. These methods are based on laser ablation inductively coupled plasma mass spectrometry and will affect clinical practice in identification and evaluation of hepatic metal disorders (for example, hereditary haemochromatosis, Wilson disease) that give rise to hepatic fibrogenesis.

## Conclusions

New international animal welfare rules will have a deep impact on fibrosis research, at least in the EU. It is thus obvious that these new regulations will affect future efforts to develop alternative animal replacement strategies. In parallel, the scientific community should improve standardisation of fibrosis models to increase the comparability of data between different laboratories with the aim of reducing animal experimentation. However, animal models are still the gold standard in fibrosis research. New, sophisticated transgenic approaches will allow investigation of specialized topics regarding fibrosis initiation, progression and resolution. Current data from these animal models prove that these findings are highly relevant and can be translated to the clinic. We hope that this review will initiate a scientific discussion of how to combine these increasing scientific innovations with enforced legal requirements.

## Abbreviations

3R: Replacement, refinement and reduction; ANIT: α-Naphthylisothiocyanate; AMA: Antimitochondrial antibody; ARRIVE: Animals in Research: Reporting *In Vivo* Experiments; CCl4: Carbon tetrachloride; DDC: 3,5-Diethoxycarbonyl-1,4-dihydrocollidine; DEN: Diethylnitrosamine; DMN: Dimethylnitrosamine; ECM: Extracellular matrix; EMT: Epithelial-to-mesenchymal transition; GSPC: Gold Standard Publication Checklist; HCC: Hepatocellular carcinoma; HSC: Hepatic stellate cell; MCD: Methionine choline–deficient diet; MFB: Myofibroblast; MMP: Matrix metalloproteinase; MMT: Mesothelial-to-mesenchymal transition; NAFLD: Nonalcoholic fatty liver disease; NASH: Nonalcoholic steatohepatitis; NK: Natural killer; PBC: Primary biliary cirrhosis; ROS: Reactive oxygen species; SOP: Standard operating procedure; TAA: Thioacetamide; TGF-β1: Transforming growth factor-β1; TIMP: Tissue inhibitor of metalloproteinase; VLDL: Very low-density lipoprotein.

## Competing interests

All authors declare that they have no competing interests.

## Authors’ contributions

RW coordinated the writing of this review, wrote sections of it and designed the figures. CL wrote part of this review, compiled Table 1 and helped to connect the individual parts of this review. FT and KS wrote individual sections of this review and helped to arrange the figures. TL, TS, DS, RT, CT and JT wrote part of this review. All authors read and approved the final manuscript.

## Authors’ information

For the Transregional Collaborative Research Centre "Organ Fibrosis: From Mechanisms of Injury to Modulation of Disease" (SFB/TRR57).
